# *DDX3X* is Epigenetically Repressed in Renal Cell Carcinoma and Serves as a Prognostic Indicator and Therapeutic Target in Cancer Progression

**DOI:** 10.3390/ijms21082881

**Published:** 2020-04-20

**Authors:** Tsung-Chieh Lin

**Affiliations:** Genomic Medicine Core Laboratory, Chang Gung Memorial Hospital, Linkou 333, Taiwan; tclin1980@cgmh.org.tw

**Keywords:** DDX3X, prognosis, renal cell carcinoma, SPINK1, digoxin

## Abstract

DEAD (Asp-Glu-Ala-Asp) box polypeptide 3, X-linked (DDX3X) is a member of the DEAD-box family of RNA helicases whose function has been revealed to be involved in RNA metabolism. Recent studies further indicate the abnormal expression in pan-cancers and the relevant biological effects on modulating cancer progression. However, *DDX3X*’s role in renal cell carcinoma (RCC) progression remains largely unknown. In this study, a medical informatics-based analysis using The Cancer Genome Atlas (TCGA) dataset was performed to evaluate clinical prognoses related to *DDX3X*. The results suggest that *DDX3X* is epigenetically repressed in tumor tissue and that lower *DDX3X* is correlated with the poor overall survival of RCC patients and high tumor size, lymph node metastasis, and distant metastasis (TNM staging system). Furthermore, knowledge-based transcriptomic analysis by Ingenuity Pathway Analysis (IPA) revealed that the SPINK1-metallothionein pathway is a top 1-repressed canonical signaling pathway by *DDX3X*. Furthermore, *SPINK1* and the metallothionein gene family all serve as poor prognostic indicators, and the expression levels of those genes are inversely correlated with *DDX3X* in RCC. Furthermore, digoxin was identified via Connectivity Map analysis (L1000) for its capability to reverse gene signatures in patients with low *DDX3X*. Importantly, cancer cell proliferation and migration were decreased upon digoxin treatment in RCC cells. The results of this study indicate the significance of the DDX3X_low_/SPINK1_high_/metallothionein_high_ axis for predicting poor survival outcome in RCC patients and suggest digoxin as a precise and personalized compound for curing those patients with low *DDX3X* expression levels.

## 1. Introduction

DEAD (Asp-Glu-Ala-Asp) box polypeptide 3, X-linked (DDX3X), also known as DDX3, is a member of the DEAD-box family of RNA helicases and has been reported to participate in several cytosolic steps of mRNA metabolism, including pre-mRNA splicing [1], gene transcription [2], RNA export [3], and protein translation [4,5,6]. The discovery of DDX3X expression alterations in various cancer types, including hepatocellular carcinoma, breast cancer, colorectal cancer, prostate cancer, and pancreatic cancer, suggests its potential role in modulating tumor behaviors [7]. Emerging evidence has further indicated the multibiological function of DDX3X in cancer cells; the oncogenic and tumor suppressive capability of DDX3X in regulating cancer proliferation, metastasis and drug resistance has been unraveled [7]. Nevertheless, evidence has shown that the complexity of DDX3X is in part due to the fact that DDX3X proteins generally do not function alone but instead act as a component in multiprotein complexes [8]. Thus, the exact function of DDX3X is determined by its interacting targets and is tumor- and/or context-dependent [9]. In addition, clinical prognosis data of DDX3X has revealed a discrepancy in several types of cancers, indicating that the clinical outcomes with respect to DDX3X remain to be explored and additional evidence is required. Renal cell carcinoma (RCC) is also termed renal adenocarcinoma, which comprises nearly 90% of kidney-derived tumors [10]. RCC corresponds to approximately 2% of malignant tumors. However, an alarming increase in incidence has been reported [11]. Although the impacts of DDX3X on many cancer types have been reported, the role of DDX3X in RCC remains largely obscure. In this study, we aimed to explore *DDX3X*’s prognostic significance and identify potential therapeutic compounds based on the transcriptomic and clinical data of RCC patients. Our results show that DDX3X is epigenetically repressed in RCC and that cancer patients displaying low *DDX3X* correlate with unfavored overall survival and tumor-node-metastasis (TNM) staging. In addition, digoxin is further characterized as a personalized and precise drug target for the malignancy treatment of those clear-cell-type RCC patients expressing low DDX3X.

## 2. Results

### 2.1. DDX3X Is Epigenetically Repressed in Tumor Tissue, and Lower DDX3X Is Correlated with Poor Overall Survival and High TNM Status of RCC Patients

**Figure 1:**
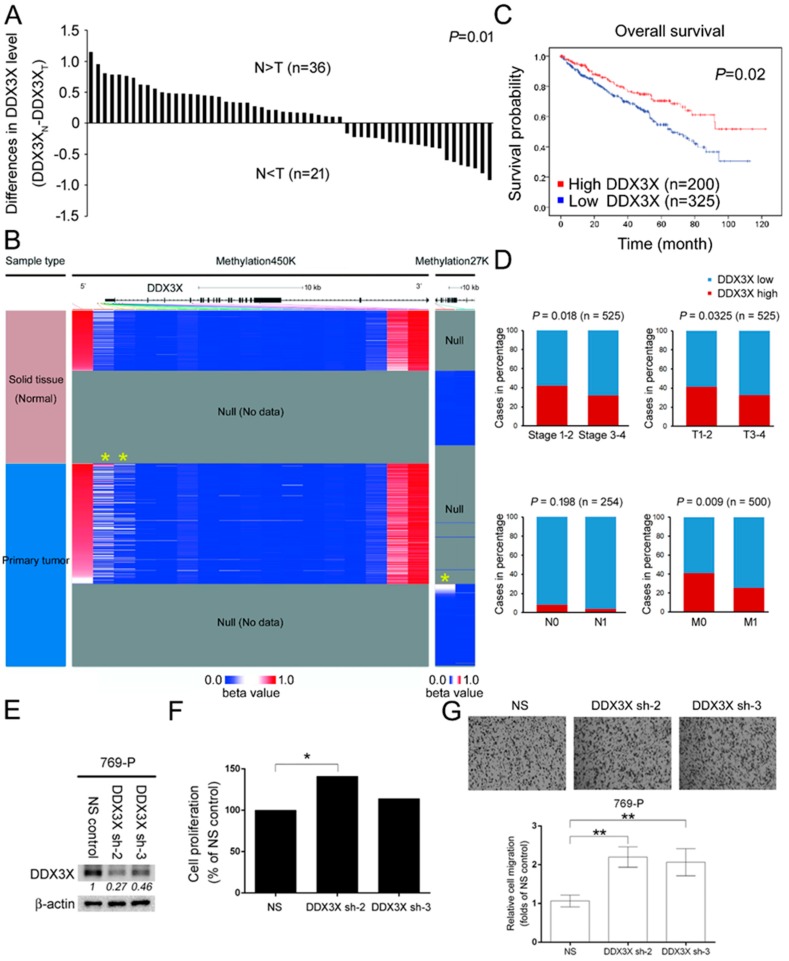
*DDX3X* is epigenetically repressed in tumor tissue, and lower *DDX3X* is correlated with poor overall survival and high tumor-node-metastasis (TNM) status in renal cell carcinoma (RCC) patients. (**A**) The expression profile of *DDX3X* in 57 matched renal clear cell carcinomas and adjacent normal tissues was compared. The gene expression profile was measured experimentally using the IlluminaHiSeq_RNASeqV2. Raw data were retrieved from TCGA database and analyzed (Dataset ID: TCGA_KIRC_exp_HiSeqV2_PANCAN). T represents tumor tissue; N represents normal adjacent tissue. The relative difference in *DDX3X* expression was obtained by *DDX3X*_N_-*DDX3X*_T_. (**B**) DNA methylation patterns in solid tissue and primary tumors of the RCC cohort were tested via Methylation450K and Methylation27K platforms and released by The Cancer Genome Atlas (TCGA). The position of the *DDX3X* protein-coding gene promoter along with the methylation pattern is indicated by a yellow asterisk. The DNA methylation fraction at a specific CpG site was calculated as beta value (β) = M/(M+U+α), where M and U are methylated and unmethylated signal intensities, and α is an arbitrary offset intended to stabilize β values where fluorescent intensities are low. (**C**) A Kaplan–Meier plot of 525 cancer patients with relatively high and low *DDX3X* levels. The case numbers in the high and low groups were determined by overall survival. (**D**) Associations of *DDX3X* expression with stage, tumor size (T1-4), lymph node (N0-N1) and distant metastasis (M0-M1) in the RCC cohort were analyzed using the χ2 test. (**E**) The DDX3X expression was silenced by the lentiviral-based transduction of specific shRNA clone 2 and clone 3 in 769-P cells. The protein levels were normalized with corresponding internal controls. The relative DDX3X protein level was shown. NS; non-silencing control. (**F**) 769-P cell numbers in indicated groups were counted by trypan blue exclusion assay after 24 h of incubation, * *p* < 0.05. (**G**) Relative 769-P cell migration upon DDX3X knockdown was evaluated by transwell assay at time point 2.5 h. The *p* values were represented by **, and considered significant (*p* < 0.01).

We previously reported the prognosis based on data from a pan-subtype of the kidney cancer cohort. The clinical significance of *DDX3X* was unobvious [7]. Interestingly, the correlation with the favored overall survival was specifically observed in the clear cell type, which is the most malignant subtype in kidney cancer, suggesting *DDX3X*’s potential role as a tumor suppressor (Table 1). To explore the clinical relevance of *DDX3X* expression levels in patients with RCC, a cohort dataset comprising 525 clear-cell-type cases, including 57 matched adjacent normal and tumor cases from The Cancer Genome Atlas (TCGA), was analyzed. *DDX3X* appeared to be more highly expressed in normal tissues than in tumors (*p* = 0.01, Figure 1A). The downregulation may in part result from the epigenetic modification that *DDX3X* promoter methylation was obviously observed in tumor samples compared with normal tissues (yellow asterisk symbols, Figure 1B). Interestingly, papillary cell carcinoma subtype appeared to have similar results of the clear cell type regarding to DDX3X’s RNA level in NT-paired sample and methylation intensity. *DDX3X* expression is higher in normal tissues as compared with the tumor samples (Supplementary Figure S1A). In addition, a significant methylation at *DDX3X* promoter region was detected in papillary cell carcinoma (Supplementary Figure S1B). However, no normal tissues were enrolled in the kidney chromophobe subtype for corresponding comparison (Supplementary Figure S1B). The Kaplan–Meier plot shows the poor overall survival of patients with lower *DDX3X* expression (*p* = 0.02, Figure 1C). In addition, univariate and multivariate Cox regression analysis revealed that low *DDX3X* level was a significant and independent predictor of poor outcome (Table 2). Furthermore, low *DDX3X* was also correlated with late disease stage, large tumor size and distant metastasis (Figure 1D). A similar trend was detected in cases with lymph node metastasis, although the result was not significant possibly due to the limited number of cases. Furthermore, DDX3X expression was silenced via a lentiviral-based transduction of two specific DDX3X shRNA clones in 769-P cells, respectively (Figure 1E). A significant increase in cell proliferation was observed in shRNA clone 2 group (Figure 1F). In addition, transwell assay was performed upon DDX3X knockdown in 769-P cells. The loss of DDX3X expression appeared to elicit cell migration capability (Figure 1G). To further test the influence of epigenetic modulation on cancer cells. A498 cells were treated with DNA methyltransferase (DNMT) inhibitor 5-Aza-2’-deoxycytidine (5-azadC) for 24 h, and a dose-dependent decrease in cell proliferation was observed (Supplementary Figure S2A). A sublethal dose of 3 μM 5-azadC was selected. The results show that 5-azadC addition caused the inhibition of A498 cell migration capability (Supplementary Figure S2B).

### 2.2. Knowledge-Based Transcriptomic Analysis Revealed that the SPINK1 Pathway Is the Top 1-Altered Canonical Signaling Pathway by DDX3X

**Figure 2:**
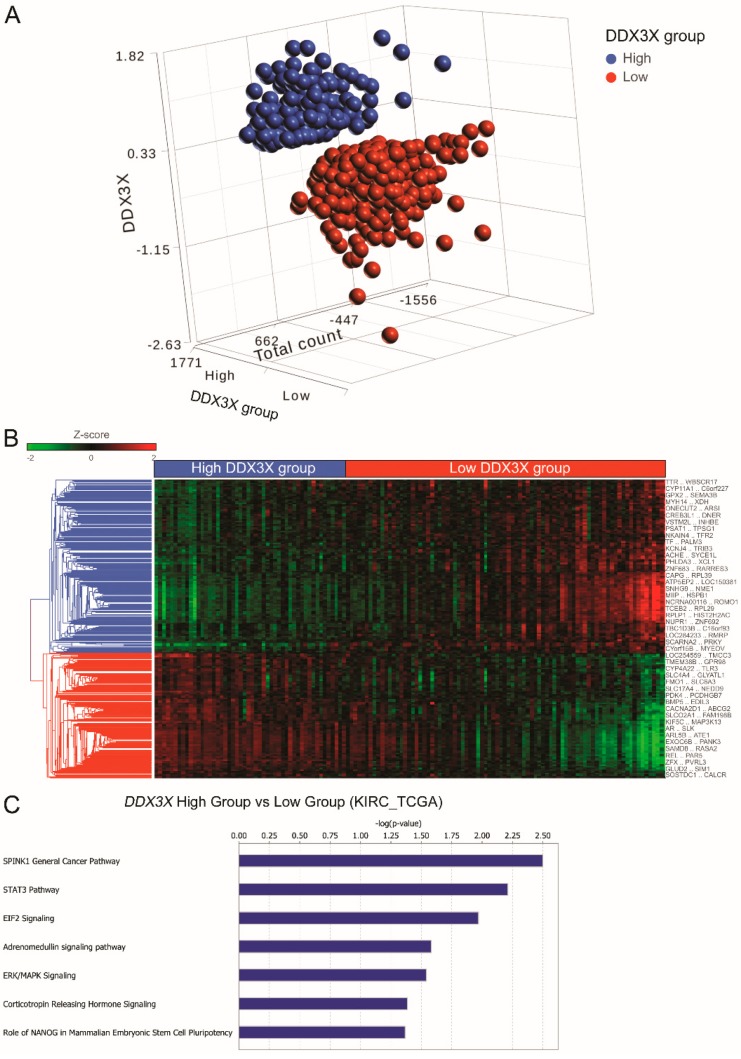

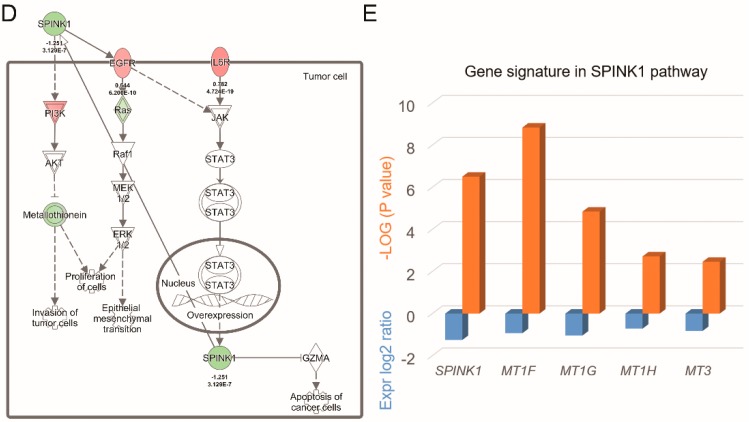
Knowledge-based transcriptomic analysis reveals that the SPINK1 pathway is the top 1-altered canonical signaling pathway by *DDX3X*. (**A**) A total of 525 cases were divided into 200 cases in the high *DDX3X* group and 325 cases in the low *DDX3X* group based on the expression level of DDX3X and association with clinical outcome. (**B**) The hierarchical clustering of gene signatures in the *DDX3X* high and low groups. (**C**) The differentially expressed gene signatures among the high versus low DDX3X group were analyzed by Ingenuity Pathway Analysis (IPA). Significant canonical pathways determined by the level of gene up- and downregulation and the amount of target overlaps in the database are listed. (**D**) The SPINK1 signaling axis is shown along with the expression ratio and statistical *p* value. The expression level was log2-transformed. Up- and downregulation are presented in red and green, respectively. (**E**) The gene targets with significant expression alterations in the SPINK1 signaling pathway are listed.

Transcriptomic and clinical data of a ccRCC cohort enrolling 525 patients were retrieved from TCGA and analyzed [12]. Patients were divided into high and low groups according to the relative *DDX3X* expression levels, and the bias was not obvious due to the similar distribution of total count in two groups (Figure 2A). We next sought to dissect the molecular mechanism of low DDX3X-associated tumor progression that leads to poor outcomes in RCC patients. RNA-Seq data were investigated. A hierarchical clustering analysis of the transcriptome by high/low *DDX3X* groups displayed a distinguished pattern on the heatmap, suggesting the merit of further studying transcriptomic alterations to justify clinical observations (Figure 2B). Gene targets with a significant differential expression were selected upon comparison of the high-DDX3X with low-DDX3X group and were further studied via the “Canonical Pathways” module of the knowledge-based Ingenuity Pathway Analysis (IPA) software. Pivotal canonical pathways were identified based on the overlap of gene targets with the IPA database, and the corresponding expression pattern was also considered by the algorithm (Figure 2C). Among the predicted pathways, SPINK1-metallothionein signaling was the top pathway with a significant correlation (Figure 2C,D). The aberrant activation of SPINK1 signaling could contribute to tumor malignancy, including increased invasion and proliferation of tumor cells [13,14,15]. Both *SPINK1* and the metallothionein gene family, including *MT1F*, *MT1G*, *MT1H,* and *MT3*, were downregulated in the comparison of *DDX3X*_high_ versus *DDX3X*_low_ (Figure 2E), suggesting *DDX3X*’s critical role in repressing RCC progression. In addition, 5-azadC treatment further led to metallothionein downregulation, which suggesting the link of DDX3X to SPINK1-Metallothionein signaling (Supplementary Figure S3). 

### 2.3. SPINK1 and the Metallothionein Gene Family Serve as Prognostic Indicators that Inversely Correlate with DDX3X in RCC

**Figure 3:**
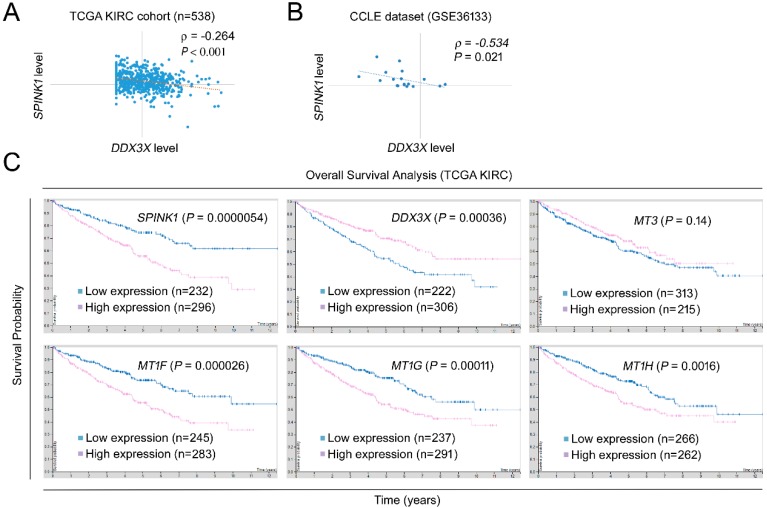
*SPINK1* and the metallothionein gene family serve as prognostic indicators that reversely correlate with *DDX3X* in RCC. (**A**) The Pearson correlations of *DDX3X* and *SPINK1* in the RCC cohort (**A**) and the CCLE dataset (GSE36133) (**B**) were evaluated. (**C**) The prognostic significance of gene targets from the SPINK1 pathway is shown. The prognostic power of overall survival was evaluated based on the mRNA expression level of the indicated targets in the TCGA RCC cohort. The data were retrieved from The Pathology Atlas (https://www.proteinatlas.org/) (access on 9 March 2020).

We alternatively investigated the impact of *DDX3X* on RCC progression by a genome-wide analysis of differential gene expression in the Cancer Cell Line Encyclopedia (CCLE) [16,17]. Importantly, a significant reverse correlation of *SPINK1* and *DDX3X* expression levels was observed in both the RCC cohort (Figure 3A) and the CCLE dataset (GSE36133, Figure 3B), suggesting the potential regulatory effect of *DDX3X* on inhibiting SPINK1 signaling activation. We further examined the clinical significance of *SPINK1* and the metallothionein gene family in RCC. The Human Protein Atlas/The Pathology Atlas is a database combining the expression profiles detected by RNA-Seq with RCC patient clinical follow-up data [18,19,20,21,22]. The data were retrieved, and we observed an association of *SPINK1*, *MT1F*, *MT1G* and *MT1H* with poor overall survival (Figure 3C). High *MT3* expression showed a marginal significance for predicting poor outcome in the RCC cohort. Taken together, the medical informatics-based analysis suggests the importance of the DDX3X_low_/SPINK1_high_/metallothionein_high_ axis as a predictor of poor prognosis for RCC patients.

### 2.4. Digoxin Reverses the Low DDX3X-Associated Gene Signature and Represses Cancer Cell Proliferation and Migration

**Figure 4:**
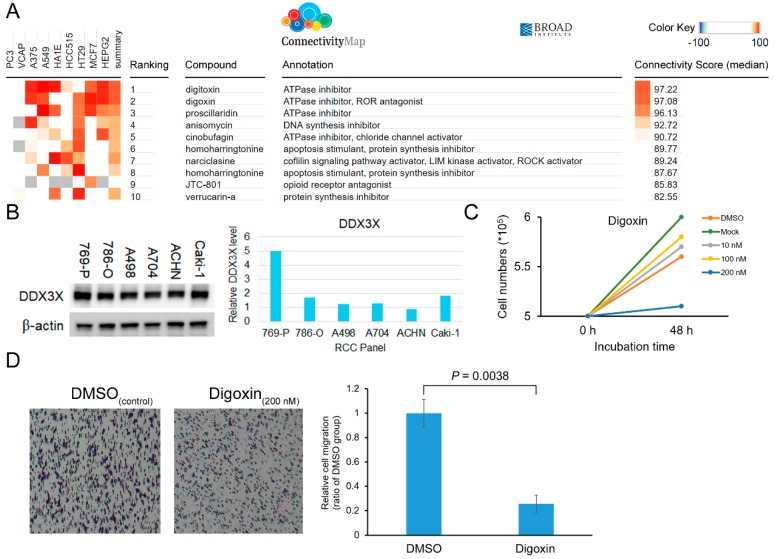
(**A**) The gene signature obtained by the high DDX3X group versus the low DDX3X group was analyzed via the L1000 platform of Connectivity Map. The drugs of the candidate were shown and ranked according to the connectivity score. (**B**) The relative DDX3X expression level was investigated in 7 clear cell subtype RCC cell lines by Western Blot. (**C**) ACHN cell numbers were counted by trypan blue exclusion assay upon 48 h of digoxin treatment. (**D**) ACHN cell migration was evaluated by transwell assay after 48 h of 200 nM digoxin treatment.

Next, we perform in silico data analysis to screen suitable compounds for inhibiting cancer progression of clear cell subtype RCC. A next generation of Connectivity Map, which contains over 1.3 million L1000 profiles has been released [23]. We then compared the DDX3X perturbation with the data in the Connectivity Map (L1000 platform) to identify potential therapeutic compounds that could reverse the gene signature observed in the RCC patient group expressing low *DDX3X*. Candidate compounds were listed according to the positive connectivity score determined after perturbation analysis (Figure 4A). Both digitoxin and digoxin showed the high similarity in gene signature compared to that in the high *DDX3X* group, and were selected for the tests of the therapeutic capabilities in cancer. Relative DDX3X expression levels were detected among a panel with 7 clear cell subtype RCC cell lines. ACHN was chosen for the lowest DDX3X level (Figure 4B). Digoxin appeared the dose-dependent reduction of cell proliferation (Figure 4C). In addition, a sublethal dose of 200 nM of digoxin was treated in ACHN cells, and the result shows the effect on decreasing cell migration as well (*p* = 0.0038, Figure 4D). However, the inhibition was insignificant by the addition of another compound, digitoxin, in ACHN cells. The results demonstrate a high throughput drug screening-based characterization of digoxin and reveal its anti-cancer progression function in RCC patients expressing low DDX3X.

## 3. Discussion

In this study, a potential drug for treating RCC patients displaying low DDX3X level was indicated. We also first uncover the prognostic significance of *DDX3X*, which is specifically correlated with good outcome in the clear cell subtype in addition to that in other subtypes. Furthermore, the potential signaling pathway altered by DDX3X was proposed. The downregulation of SPINK1 as well as metallothionein was observed in patients with high *DDX3X*. The data shown in Figure 2D further suggest the lack of SPINK1 as an upstream stimulus in RCC patients harboring high DDX3X levels, despite the upregulation of receptors including epidermal growth factor receptor (EGFR) and interleukin-6 receptor (IL6R).

The DDX3X-dependent prognosis in renal chromophobe is distinct from renal clear cell carcinoma (Table 1), suggesting the diversity regarding DDX3X’s potential impacts on tumor progression. The biological function of DDX3X is altered in part by its interactive molecules [9]. The results of a recent study showing the comprehensive immunoprofiles of renal cell carcinoma subtypes illustrated the possible reason [24]. The major difference was uncovered and shown that the c-kit protein was positively expressed in renal chromophobe, while its expression level was negative in renal clear cell carcinoma. Hence, the interplay among DDX3X and c-kit downstream signaling might be a potential target for further investigation.

Emerging evidences have also demonstrated the biological effect of DDX3X in cancer. However, the exact function of DDX3X seems to be determined by the interactive molecules and is cancer type specific [9]. Both capabilities of acting as an oncogene and a tumor suppressor have been demonstrated. The ectopic overexpression of DDX3X appears to facilitate the tumor progression of breast cancer that is through the activation of the epithelial–mesenchymal transition (EMT) process [25]. DDX3X also elicited a Snail repression-dependent glioblastoma migration [26]. Lung cancer cells expressing high DDX3X led to epidermal growth factor receptor (EGFR)-tyrosine kinase inhibitor (TKI) resistance and cancer stem cell-like phenotypes [27]. In addition, the *DDX3X* mutation was found in patients with malignant mesothelioma, indicating its potential role in tumorigenesis [28]. The recurrent mutation of *DDX3X* was also detected in Natural killer/T-cell lymphoma (NKTCL) patients and resulted in the aberrant cell cycle progression and NF-κB/MAPK signaling activation [29]. A clinical study further illustrated the correlation of high pathological stage, lymph node metastasis and unfavored prognosis in gallbladder cancer patients displaying high DDX3X levels [30]. On the contrary, oral and lung cancer patients harboring a low DDX3X expression level were reported to associate poor prognosis [31,32,33]. A study in HCT116 and U2OS cells revealed the modulation of epigenetic transcriptional and translational activation of p53 by DDX3X. DDX3X colocalized with p53 at the mitosis stage of cell cycle to ensure mitotic progression and genome stability, suggesting its role as a tumor suppressor [34]. Interestingly, both the oncogenic and tumor suppressive functions of DDX3X are also reported in the same kind of cancer. High DDX3X expression levels were detected in hepatocellular carcinoma to promote hepatocarcinogenesis [35]. In contrast, lower DDX3X was shown in hepatocellular carcinoma tissue, which was compared with adjacent non-tumor tissue. DDX3X knockdown was further proved to induce cancer cell proliferation in hepatitis virus-associated hepatocellular carcinoma [2,36]. Therefore, the conflicting roles of DDX3X not only lie between cancer types but also remain inconsistent within the same type of cancer. These controversial results emphasize the urgent need to clarify the prognostic value of DDX3X, and to unravel the molecular mechanism determining DDX3X’s oncogenic or tumor-suppressive role, especially focusing on its interactive binding molecules and downstream signaling.

## 4. Materials and Methods

### 4.1. TCGA Dataset

Gene expression in the TCGA kidney renal clear cell carcinoma (KIRC) dataset (Dataset ID: TCGA_KIRC_exp_HiSeqV2_PANCAN) was estimated by RNA-Seq (Illumina HiSeq) and retrieved for data analysis. RNA-Seq read count was normalized and log2-transformed. A total of 525 clear-cell-type cases were divided into the high *DDX3X* group (200 cases) and the low *DDX3X* group (325 cases) based on the ranking judged by the *DDX3X* expression level and the coordinated overall survival rate. HumanMethylation450 (450K) and HumanMethylation27 (27K) BeadChip assays of DNA methylation were analyzed and retrieved from UCSC Xena (https://xena.ucsc.edu/welcome-to-ucsc-xena/) (access on 20 March 2020).

### 4.2. Ingenuity Pathway Analysis (IPA)

The differential gene expression signatures in the RCC cohort were obtained after dividing the cohort into two groups displaying relatively high and low *DDX3X* levels. The gene signatures were further analyzed by Ingenuity^®^ Pathway Analysis (QIAGEN, Hilden, Germany; https://digitalinsights.qiagen.com/products-overview/discovery-insights-portfolio/analysis-and-visualization/qiagen-ipa/) according to the instructions provided. A list of relevant networks, upstream regulators and algorithmically generated mechanistic networks based on connectivity was obtained upon the comparison of Ingenuity^®^ Knowledge Database with the imported dataset. The canonical pathway analysis of IPA was also used to identify significant diseases and functions in ranking order based on the altered gene signatures.

### 4.3. Cell Culture

All human renal adenocarcinoma cell lines were purchased from the American Type Culture Collection (Manassas, VA, USA) and were the gifts from Dr. Michael Hsiao of Genomics Research Center at Academia Sinica in Taiwan. ACHN, A498 and A704 cells were maintained in MEM supplemented with 10% FBS, penicillin (100 units/mL), and streptomycin (100 µg/mL). 786-O cells were maintained in RPMI 1640 medium supplemented with 10% fetal bovine serum (FBS; GIBCO, Grand Island, NY, USA), 10 mM 4-(2-hydroxyethyl)-1-piperazineethanesulfonic acid (HEPES), 1 mM sodium pyruvate, penicillin (100 units/mL), and streptomycin (100 µg/mL). 769-P cells were maintained in RPMI 1640 medium supplemented with 10% fetal bovine serum (FBS; GIBCO, Grand Island, NY, USA), penicillin (100 units/mL), and streptomycin (100 µg/mL). Caki-1 cells were maintained in McCoy’s 5A medium supplemented with 10% FBS, penicillin (100 units/mL), and streptomycin (100 µg/mL). The cells were incubated in 95% air and a 5% CO_2_ humidified atmosphere at 37 °C. 5-Aza-2′-deoxycytidine was purchased from Sigma-Aldrich (St. Louis, MO, USA). Compound was dissolved in DMSO.

### 4.4. Cell Migration Assay

In vitro migration was investigated using transwell assays (Millipore, Bedford, MA, USA). Total of 2 × 10^5^ cells maintaining in serum-free culture medium were added into the upper chamber of the device, and the lower chamber was filled with 10% FBS culture medium. After the indicated time periods of incubation, the cells remaining on the upper surface of the filter were carefully removed using a cotton swab. The membrane was then fixed, stained and photographed. Cell motility was quantified by counting the cells in three random fields per filter.

### 4.5. Western Blot Analysis

The cells were lysed using RIPA buffer containing 50 mM Tris-HCl (pH 7.4), 150 mM NaCl, 1% Triton X-100, 0.25% sodium deoxycholate, 5 mM EDTA (pH 8.0), and 1 mM EGTA supplemented with protease and phosphatase inhibitors. After 20 min of cell lysis on ice, cell debris was removed by microcentrifugation, followed by a rapid freezing of the supernatants. The protein concentration was measured via the Bradford method. In our experiments, equivalent loads of 25–100 μg of protein were electrophoresed using an SDS-polyacrylamide gel and were then electrophoretically transferred from the gel onto a polyvinylidene fluoride (PVDF) membrane (Millipore, Bedford, MA, USA). After the blocking step with 5% nonfat milk, the membrane was then hybridized with specific primary antibodies overnight at 4 °C and was subsequently incubated in a corresponding horseradish peroxidase-conjugated secondary antibody for 1 h. The relative protein levels on membranes were visualized using an ECL-Plus Detection Kit (PerkinElmer Life Sciences, Boston, MA, USA).

### 4.6. Lentiviral-Based shRNA Production and Infection

The lentiviral shRNA constructs were purchased from Thermo Scientific (Pittsburgh, PA, USA). The experiments were performed based on our previous protocol [37]. Briefly, lentiviruses were produced by the co-transfection of a shRNA-expressing plasmid, envelope plasmid (pMD.G) and a packaging plasmid (pCMV-dR8.91) in 293T cells using calcium phosphate (Invitrogen, Carlsbad, CA, USA). The 293T cells were incubated for 18 h, and the culture medium was then removed and refreshed. The viral supernatants were harvested and tittered at 48 and 72 h post-transfection. Monolayer cells were infected with the lentiviruses in the presence of polybrene, and were further selected using puromycin.

### 4.7. Statistical Analysis

Estimates of the survival rates were processed using the Kaplan–Meier method and were compared by the log-rank test. Student’s t-test was performed for other statistical analyses. All data was shown as the mean ± S.D. The *p* values within the following levels were considered significant: * *p* < 0.05, ** *p* < 0.01, and *** *p* < 0.001.

## Figures and Tables

**Table 1:** The correlation of DEAD (Asp-Glu-Ala-Asp) box polypeptide 3, X-linked (DDX3X) with cancer patient survival.

Symbol	Cancer Type	Prognosis	Endpoint	*p* Value	Case	Dataset	Method
*DDX3X*	Kidney cancer-all subtypes	-	OS	N.S.	877	TCGA	RNA Seq
*DDX3X*	Renal clear cell carcinoma	Good	OS	0.0039	528	TCGA	RNA Seq
*DDX3X*	Renal papillary cell carcinoma	-	OS	N.S.	285	TCGA	RNA Seq
*DDX3X*	Renal chromophobe	Poor	OS	0.0028	64	TCGA	RNA Seq

Survival data was collected from TCGA. N.S.: no significance. “-”: no statistical significance. OS: overall survival.

**Table 2:** Cox univariate and multivariate regression analysis of pathological stage, TNM prognostic factors and DDX3X expression for overall survival in 500 renal cell carcinoma patients.

		Univariate		Multivariate	
Variable	Comparison	HR (95% CI)	*p* value	HR (95% CI)	*p* value
Gender	M:F	0.951 (0.694–1.303)	0.752	0.889 (0.564–1.4)	0.612
Stage	3–4:1–2	4.287 (3.089–5.949)	<0.001	3.574 (1.672–7.639)	0.001
T	T3-4:T1-2	2.992 (2.136–4.191)	<0.001	0.701 (0.334–1.47)	0.347
N	N1:N0	2.794 (1.486–5.255)	0.001	1.236 (0.638–2.392)	0.53
M	M1:M0	4.544 (3.303–6.251)	<0.001	2.263 (1.38–3.711)	0.01
*DDX3X*	High:Low	0.589 (0.42–0.826)	0.002	0.583 (0.359–0.946)	0.029

Note: Cox proportional hazards regression was used to test the independent prognostic contribution of *DDX3X* after accounting for other potentially important covariates. Abbreviation: F: female; M: male; HR: hazard ratio; CI: confidence interval.

## Supplementary Materials

Supplementary materials can be found at https://www.mdpi.com/1422-0067/21/8/2881/s1. Figure S1. DDX3X is epigenetically repressed in tumor tissue of RCC subtype: papillary cell carcinoma. Figure S2. Treatment of DNA methyltransferase (DNMT) inhibitor 5-Aza-2’-deoxycytidine (5-azadC) results in decrease of cell proliferation and migration. Figure S3. 5-azadC treatment leads to metallothionein downregulation.
