# Supplementary material for: DDX3X is Epigenetically Repressed in Renal Cell Carcinoma and Serves as a Prognostic Indicator and Therapeutic Target in Cancer Progression
Source: Int J Mol Sci. 2020 Apr 20;21(8):2881. doi: 10.3390/ijms21082881 (PMC7215876; doi:10.3390/ijms21082881)
Supplement: Supplementary file 1 [file ijms-21-02881-s001.pdf]

Supplementary Figure 1

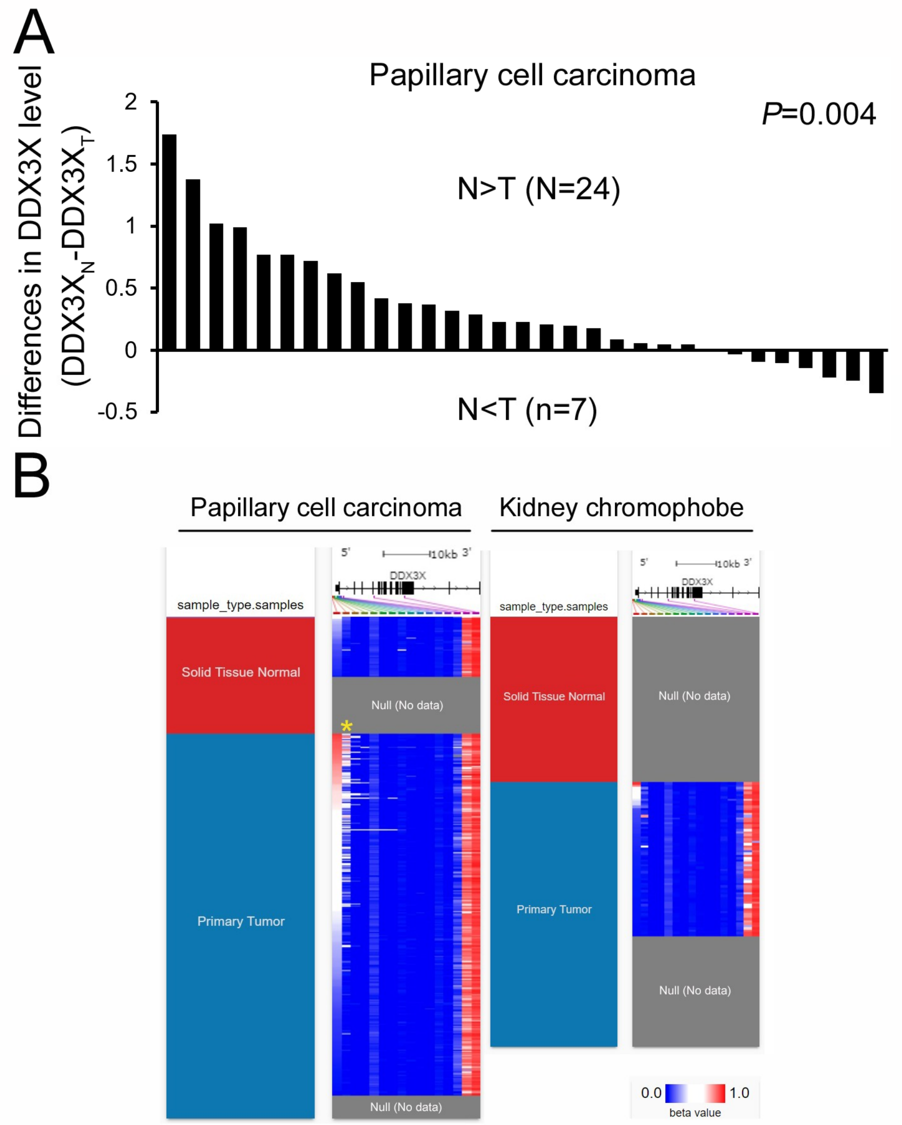

Supplementary Figure 1. DDX3X is epigenetically repressed in tumor tissue of RCC subtype: papillary cell carcinoma. (A) The expression profile of DDX3X in 31 matched renal papillary cell carcinomas and adjacent normal tissues was compared. The gene expression profile was measured experimentally using the IlluminaHiSeq_RNASeqV2. Raw data were retrieved from TCGA database and analyzed (Dataset ID: TCGA.KIRP.sampleMap/HiSeqV2_PANCAN). T represents tumor tissue; N represents normal adjacent tissue. The relative difference in DDX3X expression was obtained by DDX3XN - DDX3XT. (B) DNA methylation patterns in solid tissue and primary tumors of the RCC subtypes were tested via Methylation450K platform and released by TCGA. The position of the DDX3X protein-coding gene promoter along with the methylation pattern is indicated by a yellow asterisk. The DNA methylation fraction at a specific CpG site was calculated as beta value (β) = M/(M+U+α), where M and U are methylated and unmethylated signal intensities, and α is an arbitrary offset intended to stabilize β values where fluorescent intensities are low.

Supplementary Figure 2

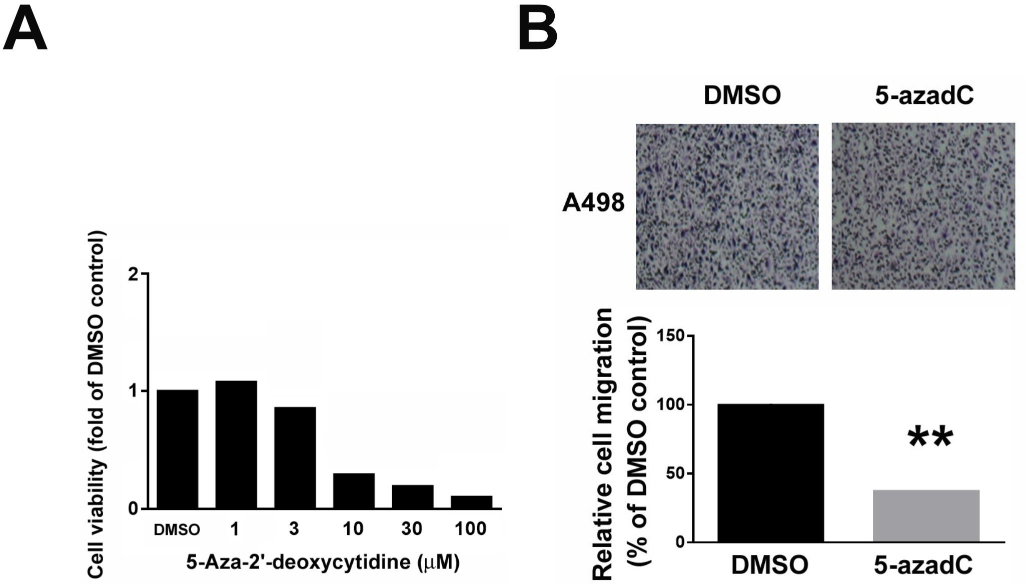

Supplementary Figure 2. Treatment of DNA methyltransferase (DNMT) inhibitor 5-Aza-2'-deoxycytidine (5-azadC) results in decrease of cell proliferation and migration. (A) A498 cells were treated with indicated doses of 5-azadC for 24h. relative cell numbers in each group were counted by trypan blue exclusion method. (B) A sublethal dose of 3 μM 5-azadC was added to A498 cells for 24h. Relative cell migration was tested via transwell assay at time point 4h.

Supplementary Figure 3

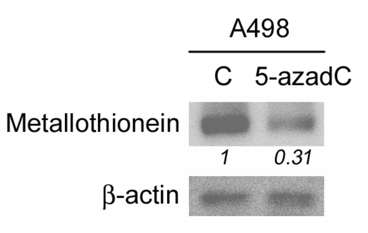

Supplementary Figure 3. 5-azadC treatment leads to metallothionein downregulation. A498 cells were treated sublethal dose of 3 μM 5-azadC for 24h. Relative metallothionein protein levels were normalized to internal controls and shown.
